# Desired but Unattained Breastfeeding: A Qualitative Exploration of Maternal Perceptions in a Cultural–Religious Context

**DOI:** 10.3390/nursrep16080291

**Published:** 2026-08-20

**Authors:** Eva Carolina Rodríguez-Huamán, María Angustias Sánchez-Ojeda, Providencia Juana Trujillo-Muñoz, Karima Mezyani-Haddu, Irene Hoyo-Guillot, Silvia Navarro-Prado

**Affiliations:** 1 Women’s Health Unit, Melilla, c/Cuerpo Nacional de Policía 4, 52004 Melilla, Spain; evrohu@correo.ugr.es; 2 Department of Nursing, Faculty of Health Sciences of Melilla, University of Granada, c/Santander 1, 52005 Melilla, Spain; ptm39@ugr.es (P.J.T.-M.); irenehoyog@ugr.es (I.H.-G.); silnado@ugr.es (S.N.-P.); 3 Melilla University Hospital, c/Luis de Ostáriz 12, 52005 Melilla, Spain; kmezyani@correo.ugr.es

**Keywords:** breastfeeding, midwifery, qualitative research, cultural factors, maternal experience, nursing

## Abstract

**Background**: The World Health Organization recommends exclusive breastfeeding for the first six months of life, with continued breastfeeding alongside complementary feeding up to two years or beyond. However, many mothers are unable to meet their breastfeeding expectations, resulting in a significant emotional impact shaped by social, cultural, and occupational factors. **Objective**: The aim of this study was to explore mothers’ perceptions and experiences of desired but unattained breastfeeding, with particular attention to its cultural–religious dimension. **Methods**: A qualitative descriptive study within an interpretivist paradigm was conducted between September 2024 and February 2025. Thirty women (15 Christian and 15 Muslim) receiving care from a primary care midwife in a Spanish city in North Africa participated. Data were collected through semi-structured interviews and analysed using inductive thematic analysis following Braun and Clarke’s approach. **Results**: Four main themes were identified: (1) emotional experiences and feelings, (2) reasons for breastfeeding discontinuation, (3) contextual and structural barriers, and (4) the influence of the social environment and support networks. Although both groups shared emotions such as guilt, frustration, and sadness, some differences were observed in how breastfeeding discontinuation was interpreted and experienced within this sample. Several Muslim participants expressed distress in relation to maternal identity and perceived maternal inadequacy, whereas several Christian participants more frequently emphasised contextual factors such as perceived lack of support or social pressure. **Conclusions**: The experience of desired but unattained breastfeeding is complex and may be shaped by cultural–religious context alongside individual, social, and structural factors. Integrating a transcultural approach into healthcare provision may improve emotional support and help reduce the burden of guilt and distress associated with breastfeeding discontinuation.

## 1. Introduction

Breastfeeding is widely recognised as the optimal form of infant feeding, with well-established nutritional, immunological, and developmental benefits [1]. Accordingly, international organisations such as the World Health Organization (WHO) recommend exclusive breastfeeding for the first six months of life, followed by continued breastfeeding alongside complementary feeding up to two years or beyond [2].

In recent years, a social environment that actively promotes breastfeeding has emerged [3], often framing it as a natural and easily established practice. This framing may contribute to the development of idealised expectations that do not always align with women’s lived experiences.

In Spain, breastfeeding rates have increased in recent years. It is estimated that 66% of infants are breastfed during the first three months, and 46.9% up to six months. However, only a minority are exclusively breastfed. According to the WHO, Europe has some of the lowest breastfeeding rates globally, with only 25% of infants receiving any breastfeeding during the first six months, and only half of these exclusively [4]. These figures highlight a persistent gap between recommendations and actual practice, suggesting the presence of complex factors that hinder the maintenance of exclusive breastfeeding.

Multiple factors may influence the discontinuation of breastfeeding, including physiological and mental health issues, work-related constraints, perceived insufficient milk supply or concerns about infant hunger, social and/or family pressures, and pharmacological treatments, among others [3,5,6]. These factors do not operate in isolation but interact to shape a multifactorial experience that may lead to early cessation of breastfeeding. Cultural and religious frameworks may also influence breastfeeding expectations and experiences by shaping beliefs about maternal roles, infant feeding, family involvement, and the social meaning attributed to breastfeeding. Cultural differences in exclusive breastfeeding rates have also been reported, with higher prevalence among women identifying with Muslim cultural–religious groups compared with those identifying as Christian [7]. However, most studies have focused on determinants of cessation, with less attention paid to how women interpret and experience unmet breastfeeding expectations, particularly from a cultural–religious perspective.

Within the context of motherhood, breastfeeding may be experienced as rewarding, while also involving physical and emotional challenges. When such challenges prevent women from meeting their initial breastfeeding expectations, they may generate emotional distress [8]. When these expectations are not fulfilled, breastfeeding discontinuation may be experienced ambivalently, involving both physical relief and feelings of guilt, frustration, and loss. In this context, an empathetic and supportive network—comprising family members, friends, and healthcare professionals—is essential to support women from physical, emotional, social, and cultural perspectives [3]. Healthcare professionals play a key role not only in providing technical support but also in offering emotionally and culturally sensitive care throughout this process. A flexible approach may help prevent women from experiencing negative feelings when breastfeeding is not successfully established or does not continue for as long as expected [9].

This study was conducted in a Spanish city located in North Africa, a unique geographical and sociocultural setting bordering Morocco, with a single regional hospital where all births are attended. This context is characterised by the longstanding coexistence of different cultural and religious groups, particularly Muslim and Christian populations, creating a distinctive multicultural environment [10]. Far from being merely contextual, this sociocultural diversity offers a particularly valuable opportunity to examine how cultural and religious frameworks shape maternal experiences of breastfeeding. In this sense, the setting provides an especially relevant context for exploring how belief systems influence the meaning and lived experience of breastfeeding.

Despite the extensive literature on breastfeeding, there is a lack of studies specifically addressing the experience of unmet breastfeeding expectations from a qualitative and culturally contextualised perspective [1,9]. This complexity, shaped by individual, social, and cultural factors, limits the generalisability of findings across contexts [9,10,11,12,13,14,15,16,17]. Therefore, this qualitative descriptive study aimed to explore mothers’ perceptions and experiences of desired but unattained breastfeeding, with particular attention to the cultural–religious context in which these experiences were narrated and interpreted.

## 2. Materials and Methods

### 2.1. Study Design and Participants

This study employed a qualitative descriptive design within an interpretivist paradigm. This approach was selected to explore how mothers described and interpreted their experiences of desired but unattained breastfeeding within their social and cultural contexts, while remaining close to participants’ accounts. An inductive thematic analysis was used to identify and interpret patterns of meaning across the dataset, following Braun and Clarke’s approach.

A total of 30 individual face-to-face semi-structured interviews were conducted, including 15 Muslim women (MW) and 15 Christian women (CW) aged between 18 and 40 years. Participants self-identified their cultural–religious affiliation, allowing the establishment of two distinct groups. The final sample comprised 30 participants, with 15 women in each cultural–religious group. Sample adequacy was considered in relation to the focused study aim, the comparative nature of the research, the balanced representation of the two groups, and the informational redundancy observed during data collection.

Participants were selected using predefined inclusion and exclusion criteria. Inclusion criteria were: women aged over 18 years who had given birth at the regional hospital, had received care in the study setting, had expressed a desire to breastfeed but had discontinued it for various reasons, and had provided both verbal and written informed consent. Exclusion criteria included: no initial intention to breastfeed, insufficient Spanish proficiency to participate in the interview without language assistance, physical and/or mental conditions preventing participation in the interview, medically contraindicated breastfeeding (maternal or neonatal), and cases involving neonatal malformations or stillbirth.

Purposive sampling was used to ensure diversity in terms of age, self-identified cultural–religious group, marital status, educational level, place of residence, self-reported socioeconomic status, and attendance at antenatal education (including type and number of sessions).

Participants were recruited through consultations with a midwife at the Women’s Health Unit in a Spanish city in North Africa. They were subsequently invited to attend a separate appointment for the interview, ensuring sufficient time and privacy to promote comfort and openness.

### 2.2. Data Collection

All individual face-to-face semi-structured interviews (Table 1) focused on participants’ personal experiences of breastfeeding. Mothers were encouraged to speak freely about topics relevant to the study, allowing them to express their thoughts, feelings, and experiences without interference or predefined responses. During data collection, the interview data were reviewed iteratively to assess informational redundancy. As data collection progressed, successive interviews increasingly reiterated experiences, perceptions, and issues already identified in earlier interviews. Informational redundancy was considered to have been observed when additional interviews no longer contributed substantively new information relevant to the study aim. This was used as an indicator of data sufficiency rather than as a formal concept of theoretical saturation. Given the comparative nature of the study, informational redundancy was considered within each cultural–religious group rather than solely across the overall sample. The assessment of informational redundancy was conducted iteratively by the principal investigator during data collection and was subsequently considered in discussions with the wider research team. The specific interview at which informational redundancy was reached in each group was not formally recorded; therefore, a precise interview number cannot be reported retrospectively. Interviews were conducted between December 2024 and February 2025. To minimise information bias, all interviews were conducted by the principal investigator, a female midwife and mother, which facilitated the establishment of trust and mutual understanding with participants. The principal investigator had previously provided midwifery care to all participants during late postpartum follow-up at the Women’s Health Unit. Participants were therefore aware of her professional role as a midwife. Her personal and family circumstances were not discussed with participants during recruitment or the interviews.

Interview duration varied according to the extent to which each participant elaborated on her experience, with an average duration of approximately 10 min and some interviews lasting up to 26 min. The interviews followed a focused semi-structured format centred specifically on participants’ experiences of desired but unattained breastfeeding. The interviews were not subject to a predetermined time limit, and none were interrupted before the participant considered her account complete. All interviews were audio-recorded using a Sony ICD-PX333 digital recorder (Sony Corporation, Tokyo, Japan) and subsequently transcribed verbatim.

All interviews were conducted in Spanish, and no interpreters were used. Adequate proficiency in Spanish was required to participate in the interviews, both to ensure direct communication between the interviewer and participants and to preserve privacy when discussing potentially sensitive personal experiences. Three women were excluded because they were unable to participate in the interview in Spanish without language assistance. Interviews were transcribed verbatim in Spanish. The quotations selected for inclusion in the manuscript were subsequently translated into English by an expert translator. Back-translation was not performed.

### 2.3. Ethical Considerations

Ethical approval was obtained on 24 December 2024 from the Research Ethics Committee (REC) of a Spanish city (Study Code: 1_672; SICEIA-2024-002861). All participants provided written informed consent prior to the interviews and were informed of their right to withdraw from the study at any time.

To ensure confidentiality, audio files, transcripts, and identifying information (name, age, and national identification number) were pseudonymised by one member of the research team and stored on password-protected devices and secure files. In accordance with the journal’s guidelines, no generative artificial intelligence (GenAI) was used in the study design, data collection, qualitative analysis, or interpretation of the findings. ChatGPT (OpenAI) (GPT-5.6 Sol, OpenAI, San Francisco, CA, USA) was used exclusively during manuscript preparation for translation review and language editing, as disclosed in the Use of Artificial Intelligence statement.

### 2.4. Data Analysis

To contextualise the interviews and enhance the interpretability of the findings, sociodemographic variables (Table 2) were entered into SPSS (version 15), which was used solely to calculate descriptive statistics for participant characteristics and not for the qualitative analysis.

**Figure 1:**
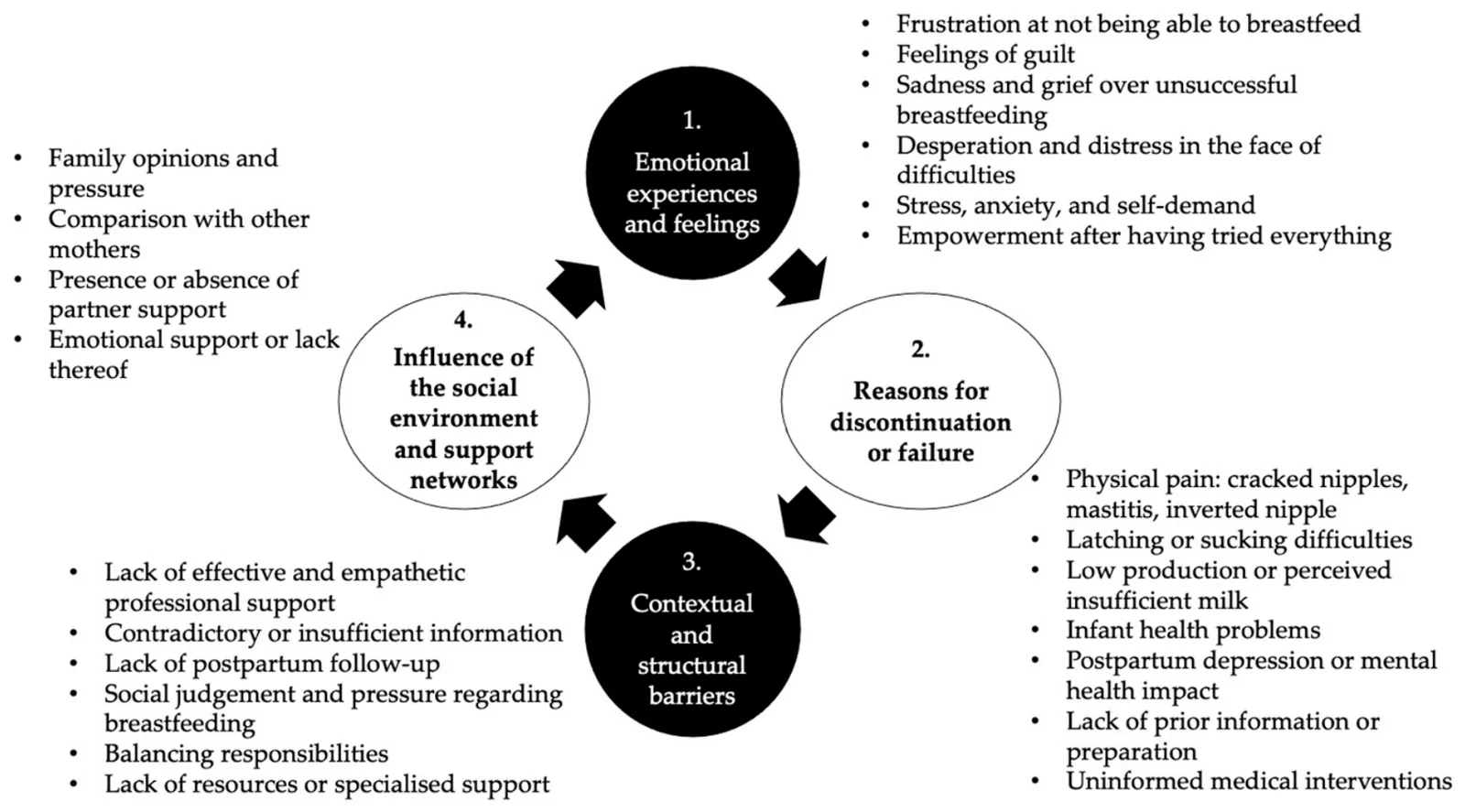
Thematic conceptual map.

Following completion of data collection, all transcripts were read in full and initially coded by the principal investigator using an inductive thematic analysis approach, following Braun and Clarke’s framework (2006). The analysis involved repeated reading of the transcripts to achieve familiarisation with the data, followed by the identification and systematic coding of meaningful units related to participants’ experiences of desired but unattained breastfeeding. Codes were subsequently examined for patterns of similarity and difference and progressively organised into candidate subthemes and overarching themes (Figure 1).

The complete transcripts, initial codes, and developing themes and subthemes were subsequently reviewed by two additional members of the research team (SNP and MSO). The three researchers discussed the interpretation and organisation of the data, and any discrepancies concerning coding or the definition and boundaries of themes and subthemes were resolved through discussion until consensus was reached. Comparisons between Muslim and Christian participants were conducted through the joint review of codes, themes, subthemes, and supporting quotations from both groups, examining both shared experiences and differences in emphasis. No formal comparative matrix or inter-rater reliability coefficient was used, as the purpose of this collaborative process was to refine the interpretation of the data through discussion and consensus rather than to quantify agreement between coders.

The research team collaboratively reviewed the data to identify similarities and differences across subthemes, with particular attention to distinctions between the two cultural–religious groups. Data collection and analysis proceeded iteratively, allowing emerging patterns to be reviewed throughout the analytical process. Data sufficiency was assessed through the informational redundancy observed during the iterative review of the interviews, as described in the Data Collection section.

### 2.5. Rigour

To ensure the trustworthiness and methodological rigour of the study, standard qualitative criteria were applied. Credibility and dependability were supported through researcher triangulation and collaborative review of the analytical process by three members of the research team, as described in Section 2.4. Interpretations were discussed until consensus was reached, allowing alternative readings of the data to be considered during the development and refinement of themes and subthemes. Confirmability was supported by verbatim recording and transcription of the interviews and by collaborative review of the analysis by the research team. The principal investigator was a midwife working in the study setting and had previously provided care to all participants during late postpartum follow-up. She was also a breastfeeding mother at the time of the study and was professionally familiar with the established benefits of breastfeeding. These characteristics were recognised as potentially relevant to her position in relation to the research topic. During the interviews, a semi-structured approach was used and participants were encouraged to describe their experiences and feelings in their own words, without the interviewer expressing her own breastfeeding experiences or personal views. During analysis, the principal investigator initially coded the transcripts, after which the complete transcripts, codes, themes, and subthemes were reviewed collaboratively with two additional researchers, allowing interpretations to be discussed and alternative readings of the data to be considered. Finally, transferability is supported by providing a detailed description of the participants’ sociodemographic characteristics, the purposive sampling strategy, and the specific multicultural setting where the study took place.

## 3. Results

The final sample comprised 30 women who experienced desired but unattained breastfeeding, of whom 15 self-identified as Christian and 15 as Muslim. No participants from other cultural–religious groups attended the consultation during the study period.

The sociodemographic characteristics of the overall sample and stratified by cultural–religious group are presented in Table 2.

Among Muslim women (n = 15), the mean age was 25.9 years (SD = 4.6). Most were married (93.3%), while 6.7% were single. Regarding educational level, 6.7% had completed primary education, 26.7% lower secondary education, 20.0% upper secondary education, 26.7% vocational training, 6.7% intermediate-level training, and 13.3% held a university degree. Socioeconomic status was predominantly medium (93.3%), while 6.7% reported a high socioeconomic status. Antenatal education had been attended by 66.7% of Muslim participants, whereas 33.3% had not.

Among Christian women (n = 15), the mean age was 26.8 years (SD = 5.7). Just over half were married (53.3%), while 46.7% were single. Regarding educational level, 13.3% had completed primary education, 6.7% lower secondary education, 20.0% upper secondary education, 20.0% vocational training, 6.7% intermediate-level training, and 33.3% held a university degree. Most Christian participants reported a medium socioeconomic status (86.7%), while 6.7% reported a low and 6.7% a high socioeconomic status. Antenatal education had been attended by 80.0% of Christian participants, whereas 20.0% had not.

The findings revealed that mothers experienced a range of difficulties throughout the breastfeeding process, with some differences observed between the two self-identified cultural–religious groups. Thematic analysis focused on mothers’ perceptions and experiences of desired but unattained breastfeeding. Four main themes were identified: (1) emotional experiences and feelings, (2) reasons for breastfeeding discontinuation, (3) contextual and structural difficulties, and (4) the influence of the social environment and support networks. Each theme comprised several subthemes. The themes, subthemes, and illustrative quotations are presented in Table 3.

### 3.1. Emotional Experiences and Feelings

This theme captures emotional expressions associated with desired but unattained breastfeeding, allowing for an exploration of how women experienced this process. The selected narratives clearly illustrate the subjective impact that emerges when breastfeeding does not unfold as expected, highlighting the symbolic weight this experience carries within the context of motherhood.

“I felt sad because I expected it to last longer (breastfeeding). I felt bad, as if I were failing as a mother. I kept thinking: ‘why can’t I feed my baby myself?’ I felt frustrated, useless, very sad.”

(Mw1)

“Terrible, it made me feel really bad, like I was a bad mother, as if I were taking something very important away from my daughter. I felt awful, and I cried all day.”

(mw5)

“It was very hard for me. I felt empty, as if I were taking something away from my baby.”

(mw9)

“Honestly, I felt terrible. Terrible because I felt I couldn’t manage something that is supposed to be natural, something all mothers do. It hurt a lot, but more than the physical pain, it was the thought that I was failing as a mother.”

(mw10)

“I felt an overwhelming sadness. I felt so guilty, as if I were choosing the easier option rather than what was best for my daughter.”

(cw1)

“Sometimes it was quite overwhelming because I would breastfeed him, and people around me would say, ‘if he asks for it so often, it’s because he’s still hungry’, so you feel like you’re not giving him enough, which was quite frustrating.”

(cw12)

“I don’t know, I felt a mix of anger and guilt. Anger because I didn’t have more support, and guilt because I thought that if I had tried harder, maybe I would have managed.”

(cw15)

“It was very hard. I felt incapable, helpless, completely overwhelmed. I cried a lot, didn’t sleep well, and everything revolved around the fact that I couldn’t breastfeed the way I wanted to.”

(cw11)

Although participants in both groups expressed intense feelings of guilt, sadness, frustration, and helplessness, some differences were observed in how these experiences were articulated within this sample. In the narratives of several Muslim participants, distress was expressed in relation to feelings of maternal inadequacy, emptiness, or difficulty fulfilling what they perceived as an expected maternal role. In the narratives of several Christian participants, contextual elements such as perceived external judgement, the social environment, and lack of support were more frequently emphasised. These patterns represent differences observed in participants’ accounts within this specific sample and should not be interpreted as characteristics inherent to Muslim or Christian women.

### 3.2. Reasons for Breastfeeding Discontinuation

This theme brings together accounts in which women explain the specific reasons that led them to discontinue breastfeeding. These reasons are not presented as isolated obstacles, but rather as complex experiences that combine physical, emotional, and contextual dimensions. The selected narratives highlight both the objective difficulties encountered and the symbolic weight surrounding decision-making in relation to infant feeding.

“They told me my baby wasn’t eating enough, that I had to give her a bottle, and I just couldn’t cope anymore with the pain and the stress.”

(MW6)

“It’s true that I was in a lot of pain, I was crying, and I had a fever, so my mother told me it was better to stop breastfeeding because it was making me ill.”

(MW8)

“I breastfed for a week, then I developed mastitis and had to go to the emergency department. They prescribed antibiotics and told me it was best to stop. My mother-in-law kept insisting that I should give formula from the first day.”

(MW11)

“The first time I stopped breastfeeding was after three days because I had severe mastitis. I had a fever, I was crying and very distressed, and I felt very frustrated, as if I wasn’t capable of doing something so basic.”

(MW12)

“I didn’t stop myself, it just stopped. It stopped because my baby wasn’t getting enough, so I had to give a bottle, and then he didn’t want the breast anymore.”

(CW7)

“It hurt so much, and I cried every time I tried. They told me it was fine to give a bottle, that the important thing was that the baby was fed.”

(CW14)

“I had an emergency caesarean section and was separated from my baby for many hours. When they brought him to me, he couldn’t latch properly, and they told me to give a bottle.”

(CW8)

“Bad, very bad because I had to stop without wanting to. I was forced by work, and it made me extremely sad. It was very hard to accept, I cried for several days. My mother told me to do what I could, but not to become obsessed.”

(CW13)

Both Muslim and Christian women described breastfeeding discontinuation as being influenced by physical factors such as pain, mastitis, or perceived insufficient milk supply, as well as by external pressures including healthcare professionals, family members, and work-related demands. However, some differences emerged in how these influences were experienced and articulated.

Within this sample, several Muslim participants described the influence of family members, particularly mothers and mothers-in-law, in relation to breastfeeding discontinuation, often alongside accounts of physical pain, mastitis, fever, or exhaustion. Several Christian participants, in turn, described circumstances related to healthcare or employment, including caesarean section, separation from the infant, difficulties with breastfeeding initiation, and return to work.

Across both groups, participants frequently described breastfeeding discontinuation as occurring in circumstances in which their initial intention to breastfeed was constrained by physical difficulties or external influences. The relative emphasis placed on family, healthcare, and work-related factors varied across participants and should be understood as patterns identified within this sample rather than as characteristics inherent to either cultural–religious group.

### 3.3. Contextual and Structural Barriers

This theme groups together accounts referring to external obstacles that interfere with the ability to sustain breastfeeding. Women describe how environmental conditions-particularly institutional and work-related- limit, hinder, or render breastfeeding unfeasible. These narratives highlight that breastfeeding is not solely dependent on individual intention but is also shaped by the structural conditions in which it takes place.

“I did try to breastfeed, but in the hospital, they didn’t explain anything to me, and when I got home I felt completely lost and no one helped me, so I started using a bottle.”

(MW2)

“(Laughs). I didn’t feel bad because it just wasn’t possible. I was alone and had to take care of my other children as well. I didn’t have time or help, and it was impossible to continue.”

(MW7)

“Terrible, I cried. It made me feel really bad. There weren’t even nurses to explain how to do it, everything was very cold and rushed, and in the end I didn’t feel encouraged to continue.”

(MW5)

“In antenatal classes everything is very general, not personal. No one says: ‘Let’s see your case’, ‘Sit down, let’s see how you’re doing’, ‘How are you managing?’ Do you understand? There’s a lot of misinformation.”

(MW15)

“I stopped because I went back to work and they didn’t give me time to express milk. Between that and the baby getting used to the bottle, it just ended.”

(CW7)

“I was in Valencia with pneumonia, I was hospitalised for a week… I was given quite a lot of medication… The doctors told me: ‘Don’t even think about breastfeeding the baby’. And it upset me because he was so small and used to the breast… It was hard for him to take the bottle.”

(CW15)

“I had a caesarean section and didn’t see him for hours. When they finally brought him to me, he couldn’t latch properly, and no one came to help me with positioning.”

(CW8)

“I wanted to continue, but in primary care they told me that since I was already using formula, it didn’t matter if I stopped completely, so I did.”

(CW10)

Both Muslim and Christian women reported structural barriers that hindered breastfeeding, including lack of support in hospital settings, unmanaged pain, and insufficient professional guidance. However, differences emerged in how these barriers were experienced and narrated.

Several Muslim participants described experiences of isolation, lack of personalised information, and perceived limitations in the support received from healthcare professionals. Their accounts included difficulties in obtaining practical guidance adapted to their individual circumstances and, in some cases, perceptions that the advice received did not adequately address their breastfeeding difficulties. Christian participants also reported insufficient professional support, particularly in relation to limited follow-up, inconsistent advice, and difficulties obtaining practical assistance when breastfeeding problems arose.

Across both groups, participants therefore described perceived limitations in healthcare support during breastfeeding, although the specific circumstances and emphasis varied across individual accounts. These findings reflect participants’ perceptions and experiences within this sample and should not be interpreted as an independent assessment of the adequacy or quality of the healthcare provided.

### 3.4. Influence of the Social Environment and Support Networks

This theme captures expressions that highlight the role played by individuals in the immediate social environment in shaping the breastfeeding experience. Partners, family members, friends, and healthcare professionals emerge as key figures who may support, influence, or even destabilise the process. The accounts illustrate how breastfeeding is a socially situated experience, shaped by advice, judgement, assistance, and interference, all of which influence both decision-making and women’s emotional well-being.

“What frustrates me is not being alone with my baby. That doesn’t bother me. What overwhelms me is when there are people around giving their opinions, like my husband, my mother-in-law and my sister-in-law. That really affects me, that’s what’s frustrating.”

(MW5)

“They saw me suffering and told me, ‘Just give a bottle and that’s it’. They didn’t really encourage me, to be honest.”

(MW3)

“I felt quite bad because breastfeeding wasn’t how they had described it to me. Everything was more difficult, my milk wasn’t coming in, the baby was crying, and I felt very insecure and nervous. I thought it was my fault. My partner told me that if I wanted to stop, I could, that he supported me.”

(MW6)

“In the end, what you need is for someone to help calm things down so you can have a moment of peace. I already had another baby at home, and it was very stressful. I didn’t have help in that sense. My partner was the main issue, actually. Grandmothers do help, but in the end, the person who needs to help you is the one who lives with you every day, and I didn’t have that support. You know? He would go to work, and it was like, ‘There you are, on your own with the two girls’.”

(MW8)

“Well… I didn’t feel bad in the end because I had a lot of support. My partner helped me a lot; he kept telling me I was doing well even when I felt I couldn’t cope.”

(CW4)

“I was very anxious, I cried all the time. My mother supported me, but she also told me that if I was suffering so much, it wasn’t worth continuing.”

(CW6)

“My husband, my mother… everyone supported me: ‘Come on, you can do it’, ‘Let’s try again’, but it just didn’t work.”

(CW9)

“Bad, very bad because I had to stop without wanting to. I was forced by work, and it made me extremely sad. It was very hard to accept, I cried for several days. My mother told me to do what I could, but not to become obsessed.”

(CW13)

Both Muslim and Christian women agreed that decisions about continuing or discontinuing breastfeeding were strongly influenced by their immediate social environment. However, differences emerged in how this influence was expressed and experienced.

Accounts from Muslim participants frequently referred to direct advice or pressure from family members, particularly mothers, mothers-in-law, and other female relatives, regarding breastfeeding continuation or the introduction of formula. Among Christian participants, narratives more often described partner support alongside advice from mothers or other relatives not to persist with breastfeeding when the experience became particularly difficult.

The immediate social environment was therefore described as an important influence on breastfeeding decisions in both groups, although the nature and intensity of this influence varied across individual accounts.

## 4. Discussion

The aim of this study was to explore mothers’ perceptions and experiences of desired but unattained breastfeeding among Christian and Muslim women within a multicultural setting. Overall, similar themes and subthemes were identified across both groups, reflecting shared physical, emotional, and contextual challenges. However, some differences were observed in how participants described and attributed meaning to these experiences. Within this sample, several Muslim and Christian participants differed in the emphasis placed on maternal identity, family and social influences, healthcare experiences, and contextual barriers. These findings suggest that cultural–religious context may contribute to shaping breastfeeding experiences alongside other individual, social, and structural factors, rather than acting as an isolated explanatory factor.

### 4.1. Emotional Experiences and Feelings

Desired but unattained breastfeeding had a profound emotional impact on participants, who described feelings of frustration, guilt, and insecurity. These experiences are consistent with previous research highlighting the influence of family and social environments on women’s emotional experiences of breastfeeding [9]. These experiences may also be intensified by the physical difficulties associated with breastfeeding, which can contribute to its discontinuation [11,12,13,14]. The emotional response to unmet breastfeeding expectations has been conceptualised in the literature as a “silent grief”, referring to emotional suffering that may remain insufficiently recognised or validated [12]. This concept provides a possible interpretive framework for participants’ accounts of guilt, frustration, isolation, and feeling insufficiently understood or supported, rather than representing a theme directly identified by participants themselves.

Although participants in both groups described guilt and frustration, some differences were observed in the way distress was narrated within this sample. Several Muslim participants placed greater emphasis on feelings related to maternal identity and perceived inadequacy, whereas several Christian participants more frequently referred to external pressures, social judgement, and perceived lack of support. These differences should not be attributed exclusively to cultural–religious affiliation. Rather, the narratives may reflect the interaction of cultural and religious frameworks with family and social dynamics, healthcare experiences, socioeconomic circumstances, and individual biographies. The findings therefore suggest that cultural–religious context may contribute to how breastfeeding experiences are interpreted, while representing only one of several interacting influences.

This emotional complexity is reinforced in other local contexts. For example, a qualitative study conducted with participants in Ourense (Spain), without distinction of ethno-religious background, revealed that the experience of breastfeeding is intrinsically challenging and characterised by emotional ambivalence. On the one hand, mothers reported a certain degree of satisfaction; on the other, feelings such as uncertainty, sadness, and even distress were predominant [1]. Similarly, in a literature review that did not differentiate by ethnicity or religion, mothers reported feelings of guilt, loneliness, and shame, which in some cases led to breastfeeding cessation and maternal depression [9].

In this regard, the findings highlight the need for healthcare professionals to recognise and validate these emotions as part of the process, avoiding normative discourses that may intensify maternal guilt.

### 4.2. Reasons for Breastfeeding Discontinuation

The literature identifies physical pain, including cracked nipples, engorgement, and mastitis [15], postpartum depression [16], and perceived low milk supply as important reasons for breastfeeding discontinuation. Negative emotional experiences have also been associated with physiological responses that may affect oxytocin release and milk production [17,18,19,20]. These factors are further exacerbated by issues such as ineffective sucking, ankyloglossia, and slow infant weight gain [21,22,23].

However, the narratives also suggested differences in the circumstances participants associated with breastfeeding discontinuation. Some Muslim participants described family members encouraging cessation in response to maternal physical discomfort, whereas some Christian participants more frequently referred to perceived limitations in healthcare support. These accounts included experiences of mother-infant separation following caesarean section or neonatal admission and the introduction of formula feeding, which participants perceived as having affected breastfeeding continuation [24,25]. These findings represent participants’ accounts of their experiences and should not be interpreted as an independent assessment of the clinical appropriateness of the interventions described. This is consistent with findings reported in previous studies [26,27]. Importantly, these patterns should not be interpreted as primarily determined by cultural–religious affiliation. Rather, they may reflect the intersection of cultural–religious identity with other social and structural determinants, including family structure, socioeconomic position, employment circumstances, and access to healthcare, some of which were not systematically examined in this study. Accordingly, the differences observed here are better understood as context-dependent patterns emerging from multiple interacting influences rather than as stable characteristics of either group.

Furthermore, participants’ accounts suggest a need for more realistic antenatal information about breastfeeding, including information on common breastfeeding difficulties, realistic expectations regarding the establishment of breastfeeding, and practical strategies for managing challenges. This interpretation is consistent with previous studies highlighting the importance of preparing women for potential breastfeeding difficulties [15,28,29]. Participants from both groups also reported receiving recommendations from healthcare professionals to introduce formula feeding in situations involving concerns about neonatal hypoglycaemia, dehydration, or weight loss [30,31]. Some participants also reported being advised to discontinue or avoid breastfeeding because of maternal medication. These accounts reflect participants’ perceptions and recollections of the clinical advice they received; the present study did not independently verify the clinical circumstances or appropriateness of individual healthcare decisions. Nevertheless, previous evidence indicates that many medications are compatible with breastfeeding [32,33,34,35], highlighting the importance of providing mothers with clear, individualised, and evidence-based information regarding medication use and infant feeding.

### 4.3. Contextual and Structural Barriers

Participants from both groups described structural barriers to breastfeeding, including insufficient healthcare support [1,36], contradictory information from healthcare professionals [1,31,32,37], and a resulting sense of frustration and isolation [38,39]. In addition, Jackson et al. [12], along with other authors [40,41], have described how the idealisation of breastfeeding as natural or instinctive may contribute to feelings of guilt and distress when breastfeeding difficulties arise. This tension was also reflected in participants’ accounts, in which difficulty achieving an experience perceived as natural or expected was associated with feelings of maternal inadequacy. Factors such as caesarean section and return to work were identified as common obstacles leading to breastfeeding discontinuation, as reported by Moreno-Ávila et al. [26]. Furthermore, antenatal education has been identified in the literature as an important protective factor [42].

Within this sample, several Christian participants placed greater emphasis on institutional factors, such as healthcare-related circumstances and return to work, whereas several Muslim participants more frequently described isolation and limitations in the support received, which may be related to their perception of a lack of understanding of their religious beliefs [43]. In the study conducted by Stein et al. [29], Muslim women breastfed for longer periods influenced by the Qur’an, although breastfeeding was often mixed, whereas Christian women showed a preference for exclusive breastfeeding. Among other differences between the groups, Christian women attended antenatal education classes more frequently [26], and experienced fewer complications during breastfeeding. Additionally, another study [27] found that Christian women were more likely to discontinue breastfeeding than Muslim women, identifying factors such as multiparity, higher religiosity, and return to work as contributors to breastfeeding cessation. These findings suggest that structural barriers do not operate uniformly but are perceived and experienced differently depending on the sociocultural context, which may influence both breastfeeding continuation and the associated emotional experience.

From a care perspective, this implies that standardised interventions may be insufficient if they do not incorporate cultural adaptation that takes into account the specific needs of each group.

### 4.4. Influence of the Social Environment and Support Networks

The findings of the present study are consistent with previous studies by Kehinde et al. [44] and Maviso et al. [45], which reported that mothers experienced considerable pressure from grandmothers in relation to traditional infant-feeding practices. These relatives often misinterpreted infant crying as a sign of hunger [37], highlighting the need to update their knowledge [31]. In contrast, the study by Xialing-Zheng et al. [46] found that the social environment acted as a facilitating factor for breastfeeding. Partner support was generally limited, reflecting traditional gender roles, in contrast with other models that emphasise both practical support (household tasks and infant care) and emotional support for the mother [31,37]. Furthermore, Campiño-Valderrama et al. [47] reported that support provided at the initiation stage did not persist throughout breastfeeding, contributing to mothers feeling alone and inadequate, as also reported by participants in this study. Similarly, Ochoa-Marín et al. [48] suggest that the high workload of nursing staff reduced the level of support and guidance provided to postpartum women, as evidenced by the offering of formula feeding.

The findings of this study also provide a cultural–religious nuance. Muslim women and Christian women agreed that decisions regarding whether to continue or discontinue breastfeeding were strongly influenced by their immediate social environment. However, MW more frequently described direct pressure from family members such as mothers, mothers-in-law, or sisters, who encouraged them to stop breastfeeding or introduce formula from the outset. In contrast, CW described a more ambiguous support network, in which partners often acted as a source of encouragement, while mothers or friends advised them not to force the process and to prioritise their well-being. Both groups acknowledged the importance of the social environment; however, among MW, statements tended to be more directive or prescriptive, whereas among CW, suggestions were more subtle or oriented towards emotional reassurance. This suggests differences in how each group receives and processes support or social pressure. The variability in the type of support received highlights that the social environment does not only act as a facilitator or barrier, but also as a factor shaping the meaning of breastfeeding and maternal decision-making.

In this regard, effective support should not be limited to the mere presence of a social network, but should also consider the quality and consistency of the messages received by the mother.

These findings also suggest that, when appropriate and with the woman’s agreement, healthcare professionals could involve influential family members, including grandmothers, in breastfeeding education and support, helping to reinforce evidence-based information while respecting the mother’s preferences and autonomy.

Within this sample, several Christian participants placed greater emphasis on institutional factors, such as healthcare-related circumstances and return to work, whereas several Muslim participants more frequently described isolation and limitations in the support received.

In comparison with a phenomenological study [26], family support was positively valued by both CW and MW. This support mainly came from mothers, partners, and sisters, although in the case of MW, the figure of the mother-in-law was particularly prominent. In addition, as MW often had larger family networks, they also received support from cousins and sisters-in-law.

### 4.5. Future Research Directions

Based on these findings, future research directions should focus on the design, implementation, and evaluation of culturally adapted antenatal and postnatal educational interventions to determine if tailored support reduces maternal distress and improves breastfeeding outcomes. Additionally, qualitative studies exploring the perspectives of healthcare professionals regarding transcultural care would be highly beneficial to identify systemic training gaps. Finally, future research should aim to include other minority ethno-religious groups present in multicultural settings to expand upon the present findings and help consolidate a truly comprehensive model of maternal support.

### 4.6. Strengths and Limitations

This study included Muslim and Christian women, who represent the most prevalent cultural groups in a Spanish city located in North Africa. However, the city is multicultural, and other minority ethnic groups coexist, such as Jewish, Roma, and Hindu communities. As these groups were not represented in the sample, the transferability of the findings to other cultural groups and geographical contexts may be limited, although the results may be relevant to similar settings.

Several methodological considerations should also be acknowledged. The relatively short duration of some interviews may have limited the depth of the data, although participants were encouraged to elaborate on their experiences through follow-up and probing questions. Recruitment from a single healthcare setting through one midwife, together with the exclusion of women unable to participate in Spanish without language assistance, may have introduced selection bias and limited the range of experiences represented. Participants’ accounts may also have been subject to recall or social desirability bias.

The principal investigator’s previous professional relationship with participants may have facilitated rapport and trust, while her professional role as a midwife and her own breastfeeding experience may also have influenced the interview interaction and interpretation of the accounts. This potential influence was addressed through review and discussion of the analysis within the wider research team but cannot be entirely excluded.

Finally, although available sociodemographic characteristics were examined separately by cultural–religious group, parity, employment status, previous breastfeeding experience, mode of delivery, and infant health conditions were not collected. These unmeasured factors may also have contributed to some of the observed differences between participants and therefore limit their attribution to cultural–religious affiliation alone.

As a strength, the sample is considered to be heterogeneous, including women with previous maternal experience and diverse socioeconomic and educational backgrounds, among other characteristics. This diversity enabled the exploration of different experiences of desired but unattained breastfeeding within the study population. At the same time, this heterogeneity may have increased variability in participants’ accounts and should be considered when interpreting the thematic patterns identified.

## 5. Conclusions

Current promotion of breastfeeding focuses primarily on its benefits, creating an idealised image that overlooks the physical and emotional complexity involved in this experience. This discrepancy between expectation and reality may lead to breastfeeding discontinuation, which in turn generates an ambivalence of emotions, combining relief with feelings of frustration and guilt. Breastfeeding discontinuation is a multifactorial phenomenon, influenced by factors ranging from physical pain to emotional support.

Therefore, this study highlights the importance of providing realistic antenatal information that reflects both the benefits and the challenges of breastfeeding, as well as the need for empathetic and personalised support. As a practice implication derived from these findings, support in multicultural settings could include culturally responsive educational resources, training healthcare professionals in culturally responsive breastfeeding care, and, when appropriate and desired by the woman, involving partners and other influential family members in breastfeeding support.

The relevance of this study lies particularly in its exploration of the cultural–religious dimension of breastfeeding experiences. Although participants in both groups described similar difficulties, some differences were observed in how breastfeeding discontinuation was experienced and interpreted within this specific sample and context. These differences should not be understood as indicating that Muslim and Christian women generally experience breastfeeding discontinuation differently and should be interpreted in light of the study context, which involved 30 participants recruited through a single healthcare setting in a Spanish city in North Africa.

In conclusion, it is essential to establish a comprehensive model of care that prioritises the mother’s physical and mental health beyond the sole objective of breastfeeding. In this regard, healthcare and social environments play a crucial role and must recognise the diversity of personal and cultural realities, ensuring respectful and woman-centred care. In other words, there is a need to move towards care models that explicitly integrate the cultural dimension into breastfeeding support, moving beyond homogeneous approaches that fail to address the diversity of maternal experiences.

## Figures and Tables

**Table 1:** Semi-structured individual interview questions.

Number	Questions
1	Please describe, in as much detail as possible, how you felt when you decided to stop breastfeeding your baby.
2	Did you experience any difficulties during breastfeeding? Which ones?
3	Why did you stop breastfeeding? (e.g., cracked nipples, pain, caesarean section, baby-related issues, postpartum depression, etc.).
4	Did you feel supported during breastfeeding by your family/friends? If not, why or how did they influence your decision to stop?
5	What positive feelings would you associate with breastfeeding? And what about negative ones (e.g., distress, loneliness, frustration, etc.)?
6	Do you feel guilty about not breastfeeding? Why?
7	Do you feel that you did everything possible to continue breastfeeding?
8	Do you think you could have done anything more to maintain it?
9	On the other hand, do you think too much importance is placed on breastfeeding? In other words, do you feel that women are sometimes pressured to breastfeed?
10	Would you like to add anything else about your breastfeeding experience?

**Table 2:** Sociodemographic variables.

Variable				
	Muslim Women		Christian Woman	
	Mean	SD ^1^	Mean	SD
**Maternal age**	n	%	n	%
	25.9	4.6	26.8	5.7
**Marital status**				
Married	14	93.3	8	53.3
Single	1	6.7	7	46.7
**Educational level**				
Primary education	1	6.7	2	13.3
Lower secondary education	4	26.7	1	6.7
Upper secondary education	3	20	3	20
Vocational training	4	26.7	3	20
Intermediate-level training	1	6.7	1	6.7
University	2	13.3	5	33.3
**Self-reported socioeconomic status**				
Low	0	0	1	6.7
Medium	14	93.3	13	86.7
High	1	6.7	1	6.7
**Attendance at antenatal education**				
No	5	33.3	3	20
Yes	10	66.7	12	80

^1^ Standard Deviation.

**Table 3:** Themes, subthemes, and illustrative quotations.

Themes	Subthemes	Textual Quotation
**1. Emotional experiences and feelings**	Frustration at not being able to breastfeed	“I felt frustrated, I couldn’t do it, I wasn’t able to, I felt incapable.”
	Feelings of guilt	“I felt guilty, I failed as a mother, I did it wrong, I regret it.”
	Sadness and grief over unsuccessful breastfeeding	“I felt sad, I cried a lot, it was like grief, I experienced it as a loss.”
	Desperation and distress in the face of difficulties	“I didn’t know what to do, I felt distressed, I was desperate, I felt like I was sinking.”
	Stress, anxiety, and self-demand	“I couldn’t sleep, anxiety was overwhelming me, I wanted to do it perfectly, I was too hard on myself.”
	Empowerment after having tried everything	“I tried everything, I did everything I could, I feel at peace, I fought until the end.”
**2. Reasons for breastfeeding discontinuation**	Physical pain: cracked nipples, mastitis, inverted nipple	“It hurt a lot, I had cracks, I couldn’t bear the pain, my breast became inflamed.”
	Latching or sucking difficulties	“The baby wouldn’t latch, would fall asleep at the breast, didn’t suck properly, choked.”
	Low production or perceived insufficient milk	“I had no milk, nothing was coming out, my breast felt empty, the baby wasn’t gaining weight.”
	Infant health problems	“The baby rejected the breast, wasn’t gaining weight, cried a lot, didn’t want to feed.”
	Postpartum depression or mental health impact	“I was very sad, everything overwhelmed me, I couldn’t cope, I had no strength.”
	Lack of prior information or preparation	“No one explained anything to me, I knew nothing, I wasn’t prepared, I thought it would be easy.”
	Uninformed medical interventions	“They gave a bottle without telling me, they separated me from my baby, they didn’t respect my plan.”
**3. Contextual and structural barriers**	Lack of effective and empathetic professional support	“No one helped me, I felt alone, they didn’t listen to me, they didn’t understand me.”
	Contradictory or insufficient information	“Everyone said something different, I was confused, they didn’t know how to guide me.”
	Lack of postpartum follow-up	“They sent me home and that was it, there were no check-ups, no one followed up.”
	Social judgement and pressure regarding breastfeeding	“They told me I had to breastfeed, they judged me, it was my duty as a mother.”
	Balancing responsibilities	“I couldn’t manage everything, I had other children, I had to return to work, it was exhausting.”
	Lack of resources or specialised support	“There were no support groups, I couldn’t find help, everything was very general, no one understood my case.”
**4. Influence of the social environment and support networks**	Family opinions and pressure	“My mother told me to give a bottle, my partner didn’t understand, everyone had an opinion.”
	Comparison with other mothers	“Others could do it, I felt inferior, they compared me, I wasn’t like them.”
	Presence or absence of partner support	“My partner wasn’t involved, said I was exaggerating, I felt alone.”
	Emotional support or lack thereof	“I had no one to talk to, I kept everything to myself, I couldn’t find comfort.”

## Data Availability

Due to the sensitive nature of the questions addressed in this study, participants were assured that their data would remain confidential and would not be shared.

## Public Involvement Statement

No public involvement in any aspect of this research.

## Guidelines and Standards Statement

This manuscript was drafted against the SRQR (Standards for Reporting Qualitative Research) [49] for qualitative research.

## Abbreviations

The following abbreviations are used in this manuscript:

CW	Christian Women
MW	Muslim Women
SD	Standard Deviation
SPSS	Statistical Package for the Social Sciences
WHO	World Health Organization
